# Effects of Selective Serotonin Reuptake Inhibitors on Interregional Relation of Serotonin Transporter Availability in Major Depression

**DOI:** 10.3389/fnhum.2017.00048

**Published:** 2017-02-06

**Authors:** Gregory M. James, Pia Baldinger-Melich, Cecile Philippe, Georg S. Kranz, Thomas Vanicek, Andreas Hahn, Gregor Gryglewski, Marius Hienert, Marie Spies, Tatjana Traub-Weidinger, Markus Mitterhauser, Wolfgang Wadsak, Marcus Hacker, Siegfried Kasper, Rupert Lanzenberger

**Affiliations:** ^1^Department of Psychiatry and Psychotherapy, Medical University of ViennaVienna, Austria; ^2^Department of Biomedical Imaging and Image-Guided Therapy, Division of Nuclear Medicine, Medical University of ViennaVienna, Austria

**Keywords:** positron emission tomography, serotonin transporter, depression, SSRI, antidepressants, connectivity, network analysis

## Abstract

Selective serotonin reuptake inhibitors (SSRIs) modulate serotonergic neurotransmission by blocking reuptake of serotonin from the extracellular space. Up to now, it remains unclear how SSRIs achieve their antidepressant effect. However, task-based and resting state functional magnetic resonance imaging studies, have demonstrated connectivity changes between brain regions. Here, we use positron emission tomography (PET) to quantify SSRI’s main target, the serotonin transporter (SERT), and assess treatment-induced molecular changes in the interregional relation of SERT binding potential (BP_ND_). Nineteen out-patients with major depressive disorder (MDD) and 19 healthy controls (HC) were included in this study. Patients underwent three PET measurements with the radioligand [^11^C]DASB: (1) at baseline, (2) after a first SSRI dose; and (3) following at least 3 weeks of daily intake. Controls were measured once with PET. Correlation analyses were restricted to brain regions repeatedly implicated in MDD pathophysiology. After 3 weeks of daily SSRI administration a significant increase in SERT BP_ND_ correlations of anterior cingulate cortex and insula with the amygdala, midbrain, hippocampus, pallidum and putamen (*p* < 0.05; false discovery rate, FDR corrected) was revealed. No significant differences were found when comparing MDD patients and HC at baseline. These findings are in line with the clinical observation that treatment response to SSRIs is often achieved only after a latency of several weeks. The elevated associations in interregional SERT associations may be more closely connected to clinical outcomes than regional SERT occupancy measures and could reflect a change in the regional interaction of serotonergic neurotransmission during antidepressant treatment.

## Introduction

The world health organization has estimated some 350 million people of all ages to suffer from major depressive disorder (MDD), which is associated with general disability and increased mortality (World Health Organization, 2015). For the treatment of MDD, selective serotonin reuptake inhibitors (SSRIs) have become the most commonly prescribed substance class (Kraft et al., 2007; Farnia et al., 2015). Their mechanism of action is based on their ability to bind the serotonin transporter (SERT), hereby inhibiting serotonin (5-HT) reuptake, thus causing an elevation in 5-HT levels in the extracellular space. However, beyond this neurochemical effect, it remains unclear how SSRIs lead to an improvement of depressive symptoms, in particular as symptom improvement occurs after a latency period of several weeks and because not all patients respond to initial treatment (Esposito and Goodnick, 2003; Kraft et al., 2007; Holsboer, 2008; Lynch et al., 2011). In addition, the SERT is involved in the pathophysiology of depression, as demonstrated by molecular imaging studies showing reduced brain SERT binding in MDD (Gryglewski et al., 2014).

In recent years, brain network analyses using magnetic resonance imaging (MRI) have evolved as an innovative approach for the characterization of complex structural and functional connections between brain areas (Bassett et al., 2008; Bullmore and Sporns, 2009; Murphy et al., 2009; Weissenbacher et al., 2009; Rubinov and Sporns, 2010). Noteworthy is also the impressive increase of resting-state fMRI (rs-fMRI) studies in the last decade, i.e., the evaluation of spontaneous low-frequency brain activations in absence of a specific task (Biswal et al., 1995, 2010). These approaches have already proven to be valuable contributions in the investigation of psychiatric disorders, as previous studies investigating MDD and SSRI treatment were able to show alterations in structural and functional brain networks between the pregenual anterior cingulate cortex and the amygdala, thalamus and striatum (Greicius et al., 2007; Anand et al., 2009; Lui et al., 2011; Zhu et al., 2012; Connolly et al., 2013; Wang et al., 2014, 2015).

Positron emission tomography (PET) studies commonly directly quantify differences in binding of molecular targets in certain brain regions, e.g., by comparing patients and healthy control subjects. Hence, the *in vivo* quantification of selected proteins may enable the identification of biological correlates underlying psychiatric disorders.

However, even if conditions or groups of subjects may differ in certain characteristics, conducting comparisons of a molecular target solely on a regional level may in some cases not be the appropriate method to capture significant differences (Vanicek et al., 2014) as it does not detect systemic or interregional changes to neurotransmitter networks. The assessment of variations within one neurotransmitter system, reflected for example by interregional changes in protein concentration, seems a promising approach. With this in mind, the acquisition of interregional associations has recently been extended to the field of molecular imaging with PET. For instance, studies of the serotonin-1A (5-HT_1A_) receptor and SERT evaluated relationships between brain regions (Hahn et al., 2010; Bose et al., 2011; Hahn et al., 2014). Moreover, these associations of 5-HT_1A_ and SERT were markedly different in patients (Hahn et al., 2014), changed after SSRI treatment (Hahn et al., 2010) and predicted SSRI treatment response (Lanzenberger et al., 2012).

The mentioned studies focused on specific interactions of the raphe nuclei in the midbrain with serotonergic projection areas. Therefore, we aimed to establish a method for the detection of molecular interregional relationships. These relationships may underline the aforementioned dysregulations proposed in connectivity, reflected by an altered SERT distribution across brain regions in MDD. Thus, unlike the comparison of protein densities in regions of interest (ROIs) and between different conditions or subject groups, we expect general interregional changes that may be associated with the reported alterations in neural circuits in psychiatric disorders, as well as the impact of treatment procedures. Similar approaches analyzing interregional metabolic relations already have been realized previously using PET and [^18^F]-fluorodeoxyglucose ([^18^F] FDG; Horwitz et al., 1984; Metter et al., 1984; McIntosh and Gonzalez-Lima, 1993; Schreckenberger et al., 1998). It could be shown that correlations of glucose metabolism between anatomically delineated areas may reflect brain functions associated with a variety of cognitive processes. Here we aim to adapt this analysis to investigate associations between regions relating to neurotransmitter properties.

Previous studies have already reported the considerable reduction of SERT availability during SSRI treatment, expectedly caused by the antidepressant’s occupation of the SERT (Lanzenberger et al., 2012; Baldinger et al., 2014). In the present study we have investigated the serotonergic circuits of patients suffering from MDD at baseline and during treatment with SSRIs. We compared correlations in SERT availability between brain regions relevant in depression. That is, despite the absolute decrease of SERT availability during SSRI treatment, we are merely interested in the relative changes between brain regions. We hypothesized that healthy subjects and patients suffering from MDD differ in the interregional relation of SERT availability between regions relevant to MDD pathophysiology. Secondly, we expected a significant change in the interregional relation of SERT availability after SSRI treatment in the MDD group.

## Materials and Methods

### Subjects

Data from 19 subjects (13 female, age range 27–54 years of age, 42.26 ± 7.84) suffering from MDD which has been included in previous publications was analyzed (Lanzenberger et al., 2012; Baldinger et al., 2014; Hahn et al., 2014). In addition, data of 19 healthy controls (HC; 6 female, age range 27–54, 37.58 ± 8.28, mean ± SD) were analyzed for comparison. The groups differ in gender distribution (*p* = 0.023), but not in age (*p* = 0.082). However, since the latter result is marginal significant, we controlled for both, age and gender in the analyses to exclude any possible influence of these variables on the overall outcome. Psychiatric disorders were assessed using a Structured Clinical Interview (SCID) for DSM-IV diagnose and a 17-item Hamilton Depression Rating Scale (HAM-D). Prior to PET measurements the patients underwent neurological and physical examinations, consisting of an electrocardiogram, a routine blood examination, a urine drug test and, in women, a urine pregnancy test. Exclusion criteria were drug abuse, medication intake preceding the PET measurements within a period of 3 months (4 months for fluoxetine) and a HAM-D score of <16 in MDD patients. All subjects provided written informed consent after briefing and complete description of the study. The study was approved by the Ethics Committee of the Medical University of Vienna and performed according to the Declaration of Helsinki.

### Study Design and Treatment

**Figure 1:**
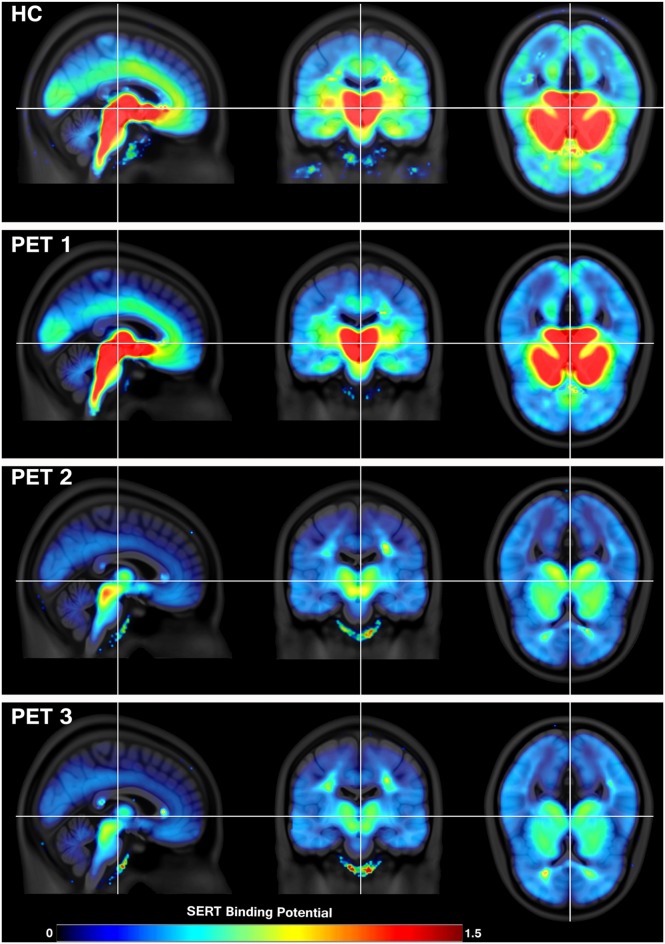
**Serotonin transporter (SERT) availability in healthy controls (HC**; ***N* = 19) and in patients with major depressive disorder (MDD; *N* = 19) during treatment with selective serotonin reuptake inhibitor (SSRI).** Positron emission tomography (PET) 1 shows the condition at baseline, PET 2 6 h after a single oral intake of SSRI and PET 3 after at least 3 weeks of daily SSRI treatment. The decrease in SERT availability indicates SERT occupancy by SSRIs during therapy, which is especially visible in brain stem, subcortical regions and the cingulate cortex. The color table indicates SERT availability from low (blue) to high (red) measured in binding potential (BP_ND_). Crosshair marks the corresponding location in sagittal, coronal and axial view (from left to right).

In this longitudinal study design, patients underwent three PET measurements: first at baseline, second within 6 h after the administration of an oral SSRI dose, and the third measurement after a minimum of 3 weeks (mean time ± SD, 24.73 ± 3.3 days) of daily oral SSRI treatment. The study medication was citalopram (R, S-citalopram, 20 mg/day, nine subjects; Lundbeck A/S, Denmark) or escitalopram (S-citalopram, 10 mg/day, 10 subjects), which constitute frequently prescribed SSRIs that are administered to millions of patients. SERT binding potential (BP_ND_) at baseline, after first and after at least 3 weeks of daily SSRI intake in patients is shown in Figure 1. HC were measured once at baseline (Figure 1).

### Positron Emission Tomography

PET measurements were performed using a GE Advance full-ring scanner (General Electric Medical Systems, Milwaukee, WI, USA) in 3D mode at the Department of Biomedical Imaging and Image-guided Therapy, Division of Nuclear Medicine of the Medical University of Vienna. For tissue attenuation correction a transmission scan of 5 min was carried out with ^68^GE rod sources. PET scans started as [^11^C]DASB was administered as a bolus injection and total acquisition time was 90 min, split into 15 × 1 min and 15 × 5 min time frames (30 time frames in total). Images were measured in kBq/ccm. Reconstruction occurred in 35 transaxial section volumes (128 × 128) with an iterative filtered backprojection algorithm (FORE-ITER) with a spatial resolution of 4.36 mm full-width at half maximum (FWHM) next to the center of the field of view (Lanzenberger et al., 2012).

### Serotonin Transporter Quantification

PET images were between-frame motion-corrected and summed images were spatially normalized to a [^11^C]DASB specific template in stereotactic Montreal Institute (MNI) space using SPM8 (Wellcome Trust Centre for Neuroimaging, London, UK^1^). The multilinear reference tissue model (MRTM2; Ichise et al., 2003) implemented in PMOD image analysis software, version 3.509 (PMOD Technologies Ltd., Zurich, Switzerland^2^) was used for the SERT BP_ND_ quantification, with cerebellar gray matter as the reference region and thalamus as the receptor-rich region. SERT availability is quantified by the BP_ND_. This binding potential compared to the nondisplaceable uptake, is defined as (V_T_−V_ND_)/V_ND_ (unitless). V_T_ and V_ND_ denote the volume of distribution in the tissue and in the nondisplaceable compartment, respectively (Innis et al., 2007).

### Regions of Interest

ROIs highly relevant in depression and SSRI treatment were selected based on both, published literature and acceptable signal to noise ratio (SNR) for SERT quantification. These ROIs mainly comprised subcortical regions, i.e., thalamus (Anand et al., 2005; Lui et al., 2011), putamen (Tao et al., 2013; Meng et al., 2014), caudate nucleus (Kim et al., 2008; Pizzagalli et al., 2009), globus pallidum (Anand et al., 2005), midbrain including dorsal and median raphe nuclei (Lanzenberger et al., 2012; Pandya et al., 2012), hippocampus (Lui et al., 2011; Sheline, 2011), and amygdala (Drevets et al., 2002; Veer et al., 2010; Lui et al., 2011; Gong and He, 2015), as well as cortical regions, i.e., the anterior cingulate cortex (ACC; Anand et al., 2005; Sheline et al., 2010; Lui et al., 2011; Pizzagalli, 2011; Gong and He, 2015) and the insula (Veer et al., 2010; Jin et al., 2011; Lui et al., 2011; Connolly et al., 2013; Tao et al., 2013). Except the midbrain, all regions were delineated using the Harvard-Oxford probabilistic atlas and averaged for both hemispheres.

### Statistical Analysis

To test for normality of the BP_ND_ values, a Shapiro-Wilk-Test was conducted, which was significant for two variables (data not shown) and due to a sample size of <20, all correlations were calculated using Spearman’s rank correlation.

Molecular relation is here defined as correlation of the SERT density between brain regions, similar to “functional connectivity” in fMRI. However, functional connectivity refers to the temporal coupling of brain regions, whereas for neurotransmitter PET no time sequences are correlated, but molecular density quantities per region pair over the entire group/condition. Correlation matrices were created by calculating Spearman’s rank correlation coefficient (rho; *ρ*) for each ROI pair over all subjects. To exclude the influence of potentially confounders, the variables age and gender were included as covariables into the partial correlation. This was done separately for each group and time point, i.e., PET 1 (at baseline), PET 2 (6 h after first treatment) and PET 3 (after at least 3 weeks of treatment), respectively. 3D volume images were generated using the Brain Net Viewer^3^ (Xia et al., 2013).

For the assessment of statistically significant differences in correlations, a 10,000-fold permutation test was performed. For the longitudinal analysis we assured that the measurements from every subject were separated into different conditions (i.e., time points) for each permutation, hence each subject was only assigned once to each condition. For overall comparison the resulting correlation matrices were transformed with Fisher’s r-to-z-transformation. A false discovery rate (FDR) correction with *p* < 0.05 was conducted, based on the number of correlations, using the Benjamini-Hochberg method for multiple comparison.

## Results

### Changes in Interregional Molecular Relation with Treatment in MDD Patients

**Figure 2:**
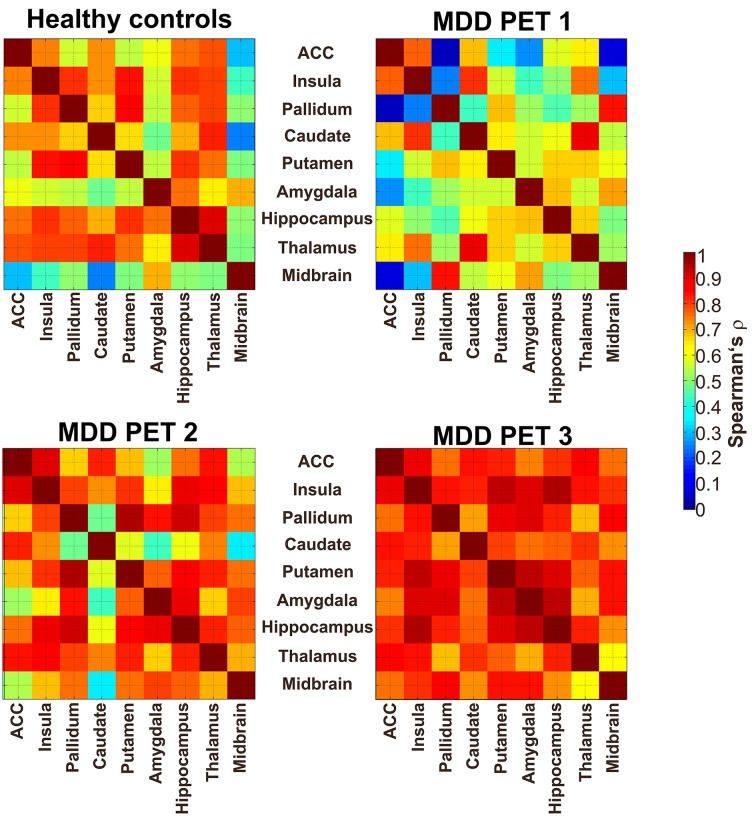
**Interregional SERT correlation matrices between nine regions of interest (ROIs).** The upper left map shows the correlation (Spearman’s *ρ*) of SERT binding in HC. The upper right map displays the condition of depressive patients at baseline (PET 1; unmedicated), the lower left and lower right maps show the SERT availability after 6 h (PET 2) and after 3 weeks of oral SSRI treatment (PET 3), respectively. ACC, anterior cingulate cortex; the color table indicates the molecular interregional relation between regions, given in Spearman’s rho (*ρ*).

**Figure 3:**
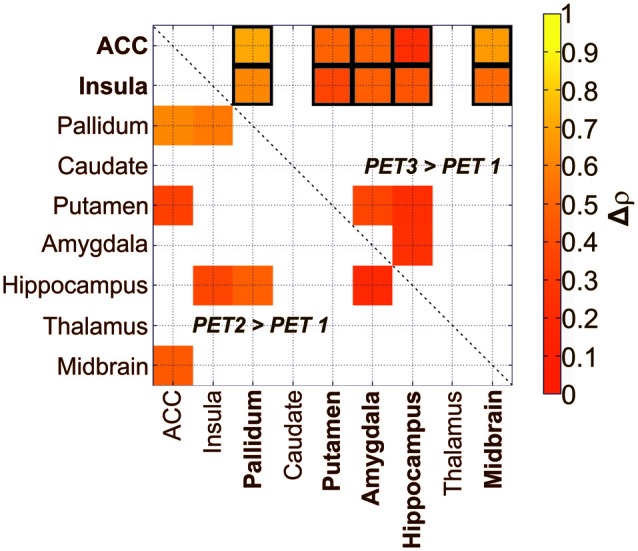
**Treatment-induced changes in the relation of SERT availability in depressive patients after administration of SSRI in two different treatment conditions (PET 2, PET 3) compared to baseline (PET 1).** The lower triangle denotes significant increases in relations after a single oral SSRI dose in depressive patients (*p* < 0.05; uncorrected). The upper triangle shows significant increases after 3 weeks of treatment (*p* < 0.05; uncorrected). Framed squares indicate changes which remain significant after FDR correction for multiple comparison at *p* < 0.05. ACC, anterior cingulate cortex; the color table indicates changes in correlation coefficients (Δ*ρ*).

**Figure 4:**
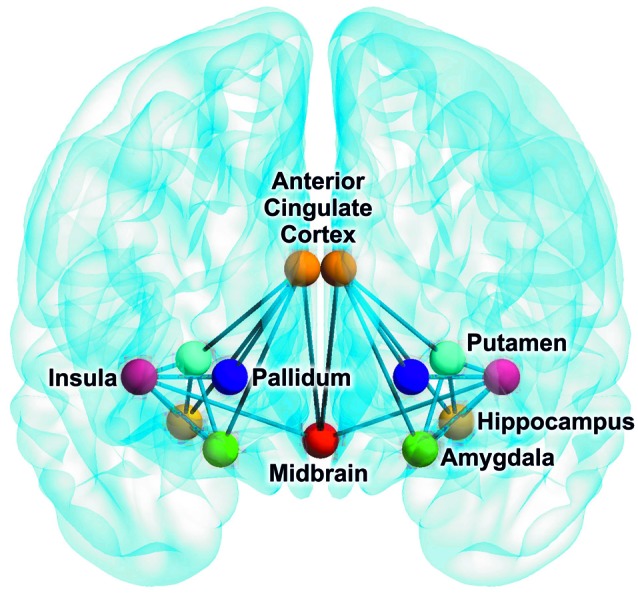
**Brain network indicating an increase in SERT relations of depressive patients after 3 weeks of SSRI treatment compared to baseline (FDR corrected).** The brain image was created with the BrainNet Viewer (http://www.nitrc.org/projects/bnv).

Interregional SERT correlation matrices for each group and time point can be seen in Figure 2. Derived from the permutation tests, differences in interregional correlations of SERT BP_ND_ were observed in several region pairs between PET 1 and PET 2 only at *p* < 0.05 uncorrected. Here, an increase was present in the molecular association of the pallidum, putamen, insula, ACC, midbrain, hippocampus and amygdala (see Table 1, Figure 3), however, without reaching significance after correcting for multiple comparison. For all of the ROI pairs associated with the ACC and insula at PET 1 vs. PET 2, except for amygdala-pallidum, the strength of correlations further increased at PET 1 vs. PET 3, such that changes seen in this comparison were significant after correction for multiple comparisons (*p* < 0.05; FDR corrected). Furthermore, additional significant and corrected correlations emerged. A significant increase in molecular relation was predominantly observed for correlations involving the ACC and insula in conjunction with the amygdala, midbrain, hippocampus, pallidum and putamen (see Table 2, Figures 3, 4).

**Table 1:** **Treatment-induced changes in the interregional molecular relation of serotonin transporter (SERT) availability in patients with major depressive disorder (MDD)**.

Before treatment compared to first treatment (PET 1–PET 2)	
ACC	Midbrain (+0.45), pallidum (+0.61), putamen (+0.34)
Hippocampus	Amygdala (+0.20), insula (+0.37), pallidum (+0.47)
Insula	Pallidum (+0.56)

*Changes are based on baseline Positron emission tomography (PET 1) compared to SERT availability after a single oral dose of a selective serotonin reuptake inhibitor (SSRI; PET 2) (values in parenthesis are differences in Spearman’s ρ; *p* < 0.05, uncorrected)*.

**Table 2:** **Treatment-induced changes in the interregional molecular relation of SERT in patients with MDD, based on the comparison of baseline (PET 1) to the SERT availability after 3 weeks of daily administered selective serotonin reuptake inhibitor (SSRI) treatment (PET 3)**.

Before treatment compared to ongoing treatment (PET 1–PET 3)	
ACC	Amygdala (+0.49)*, hippocampus (+0.24)*, midbrain (+0.67)*, pallidum (+0.71)*, putamen (+0.49)*
Insula	Amygdala (+0.47)*, hippocampus (+0.43)*, midbrain (+0.51)*, pallidum (+0.61)*, putamen (+0.36)*
Hippocampus	Amygdala (+0.24), putamen (+0.23)
Putamen	Amygdala (+0.36)

*Differences marked with an asterisk are significant after FDR correction (values in parenthesis are differences in Spearman’s rho; *p* < 0.05, uncorrected)*.

### Differences in Interregional Molecular Relations between Healthy Controls and Patients at Baseline

The comparison of MDD at baseline and HC revealed differences in relations only at *p* < 0.05 uncorrected. Involved regions are the hippocampus, insula, thalamus, midbrain and pallidum (see Table 3). After correction for multiple comparison, there was no significant interregional correlation left.

**Table 3:** **Differences in interregional molecular relation of SERT availability between healthy subjects and patient suffering from MDD (values in parenthesis are differences in Spearman’s rho; *p* < 0.05, uncorrected)**.

Healthy subjects compared to patients with major depressive disorder at baseline (PET 1)	
Hippocampus	Thalamus (−0.23)
Pallidum	Insula (−0.59), midbrain (0.34)

## Discussion

### Patients with Major Depressive Disorder during Treatment

In the current study we compared correlations in SERT availability between brain regions relevant in depression. Correlations of the ACC and insula with amygdala, midbrain, hippocampus, pallidum and putamen increased significantly after 3 weeks of SSRI treatment. These results suggest that an interregional rearrangement of SERT availability may contribute to SSRI treatment effects in MDD patients. The fact that a portion of these elevations tend to be present already after 6 h of treatment, may reflect a stabilization of these relations after continuation of SSRI treatment. These results parallel the chronological pattern seen in clinical improvement of MDD symptoms, which often requires several weeks of treatment, whereas only subtle changes can be detected in the initial phase (Taylor et al., 2006).

A number of fMRI studies investigated the influence of SSRIs on activity and functional connectivity. Reduced neural activation in the amygdala was found with fMRI when MDD patients were exposed to emotional, i.e., fearful and sad faces, following 8 weeks of antidepressant treatment (Sheline et al., 2001; Fu et al., 2004; Harmer and Cowen, 2013). Further effect of SSRI treatment could also be seen in the striatum and cortical regions, such as the pregenual anterior cingulate cortex (Fu et al., 2004). Furthermore, when investigating the functional connectivity in response to affective facial expressions, Chen et al. (2008) found a significantly increased coupling between the amygdala and the cingulate cortex, thalamus and striatum, in association with SSRI treatment. Using a similar paradigm with affective stimuli, treatment with venlafaxine (5-HT–norepinephrine reuptake inhibitor) affected the activation of the left insula already after 2 weeks of treatment (Davidson et al., 2003). To explore the presence of biomarkers to predict treatment outcomes with SSRIs, Miller et al. (2013) exposed unmedicated MDD patients to an emotional word processing fMRI task, following an 8 weeks treatment with escitalopram. They reported an association between lower activation prior to treatment in response to negative words in midbrain, dorsolateral prefrontal cortex (PFC), insula, middle frontal cortex, premotor cortex, ACC, thalamus as well as caudate, and preferable treatment outcomes.

Although the present study could not reveal significant correlations in all of the aforementioned regions, at least a tendency for the most of these was also found in SERT associations. Of those, the ACC and insula were involved in all of the significant correlations. Interestingly, a number of these correlations appear already after 6 h, although not significant at this point. Using rs-fMRI and seed based connectivity analysis, McCabe and Mishor (2011) investigated the effect of citalopram on human brain circuits and selected several seed regions, including the right amygdala and the subgenual cingulate cortex. Although they were not able to show any differences in mood compared to a placebo group, they revealed a reduced functional connectivity between the amygdala and the ventral medial PFC, when using the amygdala as seed region (McCabe and Mishor, 2011). Due to the low SNR in the cortical areas in our study using this PET radioligand, the PFC had to be excluded from the analysis. Further, it was also demonstrated that the resting state functional connectivity between the left dorsal nexus (dorsal medial PFC) and the left hippocampus was reduced after SSRI treatment (McCabe et al., 2011).

Moreover, not only the functional connectivity, but also changes in the regional glucose consumption are of interest. In a PET study assessing the total glucose metabolism with [^18^F] FDG, a general shift in glucose metabolism was observed with SSRI treatment, namely, an increased glucose metabolism in cortical areas, such as the dorsolateral, ventrolateral, medial prefrontal and parietal cortex, as well as in the dorsal ACC. On the other hand, the left insular cortex, hippocampus and parahippocampal regions showed a decreased consumption after an SSRI treatment period of 6 weeks (Kennedy et al., 2001). These shifts underline the possibility of a “normalization” effect in brain regions due to SSRI treatment that may also be driven by the alterations in SERT densities across regions.

Our current findings suggest that the therapeutic effect of SSRI treatment is mediated by rebalancing SERT in cortical and subcortical areas. In this study interregional changes occurred among the insula and ACC, in association with the midbrain, amygdala, hippocampus, pallidum and putamen. In the light of the present results, we propose that the changes in SERT relations may contribute to a better understanding of the delayed antidepressant effects during SSRI treatment, which may be reflected and influenced by a delayed adjustment of the relationship between interregional SERT densities.

### Patients with Depression vs. Healthy Control

We compared the SERT interregional relations in depressed patients at baseline with those of HC. A recent meta-analysis revealed reduced SERT availability in MDD and highlighted the impact of symptom heterogeneity, which might provide an explanation for contradictory results, when investigating the SERT in MDD patients (Gryglewski et al., 2014; Spies et al., 2015). In our comparison a tendency towards decreased SERT correlations in MDD was observed mainly for pallidum, insula and ACC. Although these are not significant after FDR correction, they contribute to our insight on differences in SERT binding in depression on a network level. Interestingly, the relations pallidum-insula and pallidum-ACC are among those elevations occurring after 3 weeks of SSRI treatment. Veer et al. (2010) reported a decreased functional connectivity of the amygdala and left insula with other regions in a whole brain network in depressed subjects. This finding may reflect the impaired ability of depressed patients to regulate negative emotions, a process in which the amygdala has shown substantial involvement (Johnstone et al., 2007; Veer et al., 2010). Further fMRI studies reported the involvement of the amygdala, pallidostriatum, medial thalamus and insula during the exposure of negative vs. neutral stimuli in patients with depressed subjects compared to HC (Anand et al., 2005), as well as frontal gyri, ACC and thalamus (Teasdale et al., 1999). It is known that the insula, ACC, temporal pole and amygdala comprise regions which are involved in emotional perception and regulation (Pessoa, 2008), as well as the medial thalamus and hypothalamus (Alexander et al., 1990; Phillips et al., 2003).

### Limitations

One limitation of this study is that we did not differentiate between first and recurrent depressive episodes in the MDD patient group. It has been previously proposed that repeated occurrences of episodes may impact on functional connectivity patterns (Veer et al., 2010) and thus deteriorate the clinical picture. However, a recent study investigating the antidepressant efficacy of pre-adult onset compared to adult-onset MDD also did not find differences regarding response, remission or tolerability of antidepressant drugs (Sung et al., 2013). The consideration of the overall treatment response or differentiation of SSRI medication type (R,S-citalopram vs. S-citalopram) might also affect the present findings. Another concern worthy to be mentioned is the relatively low number of subjects. As a consequence, the limited sample size may cause increases in false negative results, which should be improved in future studies. Nevertheless, the reported results withstood correction for multiple comparisons using the Benjamini-Hochberg method, which adequately controls for false positive findings.

Therefore, a sample size with a minimum of subjects per group is required to maintain statistical power in the application of the permutation test procedure. Thus, the results presented here were not further differentiated by treatment response outcomes, leading to even smaller group sizes. However, a less heterogenic but more extensive patient group could contribute to highlight these differences even more clearly. Further, the consideration of all brain regions, including cortical regions, in this analysis would have allowed to form more global statement in terms of interregional effects on SERT binding. The low SNR due to the sparse SERT density in the most cortical regions although, urge to focus on those regions that show a high binding. However, according to the design of the present study, no evidence can be provided if the elevated correlation of BP_ND_ between regions results from an overall decrease of interregional differences due to SERT occupancy. The observation of elevated correlations of BP_ND_ between regions may be attributed to this effect, given preserved inter-individual differences in BP_ND_. Finally, the outcomes on interregional relations presented here were determined on group level. Future studies investigating changes in interregional relations based on dynamic PET will enlighten if changes occur also in single subjects.

### Conclusion

In the present study we were able to detect changes in interregional correlations of SERT BP_ND_ with SSRI treatment in MDD patients, towards a significant increased rearrangement of SERT availability. This finding underlines the concept of interregional changes, rather than mere focal modifications, induced by SSRIs. Our results hereby contribute to a better understanding of SSRI treatment effects.

## Author Contributions

GMJ designed the methods, analyzed and interpreted the data and wrote main parts of the article. PB-M assisted the measurements and contributed to the study design. CP synthetized the radioligand and edited the manuscript. Support for the statistical implementation was given by GSK. TV assisted the measurements and contributed to the methods of the manuscript. AH gave major technical support, conceptual advice for the methodology and edited the manuscript. GG helped to develop the methodology and edited the manuscript. Advice in all medical concerns and contribution to the discussion and limitations was given by MHi. MS performed the literature search and wrote parts of the discussion. TT-W administered the radioligand and designed the measurements. MM gave technical support and developed the radioligand together with WW, which also planned the production. MHa provided the facilities for the radioligand synthesis and gave conceptual advice. SK supervised the entire experiment and patient care. RL developed the concept of the research question, provided funding and revised the manuscript. All authors discussed the results and implications and commented on the manuscript at all stages.

## Conflict of Interest Statement

The authors declare that the research was conducted in the absence of any commercial or financial relationships that could be construed as a potential conflict of interest.

## Footnotes

^1^http://www.fil.ion.ucl.ac.uk/spm

^2^http://www.pmod.com

^3^www.nitrc.org/projects/bnv
